# What makes a tree weep?

**DOI:** 10.1093/plphys/kiae161

**Published:** 2024-03-15

**Authors:** Nicola Trozzi

**Affiliations:** Assistant Features Editor, Plant Physiology, American Society of Plant Biologists; John Innes Centre, Norwich Research Park, Norwich, NR4 7UH, UK; Department of Plant Molecular Biology, University of Lausanne, CH-1015 Lausanne, Switzerland

Plants come in many shapes and sizes, from tiny mosses to large trees. A common feature among plants is their upright posture, a natural response to gravity. Roots grow down into the soil in search of nutrients and stability, while shoots grow upward, a process known as negative gravitropism. Many tree species develop a complex branching system to maximize light capture for photosynthesis, which requires the formation of tension wood—a specific anatomical adaptation to cope with gravity. Species such as magnolia (*Magnolia acuminata*) or poplar (*Populus tremula*) have branches that grow upward. However, some species, like the weeping willow (*Salix babylonica*) and Japanese apricot (*Prunus mume*), are known for their gracefully drooping branches. Despite recognizing the impact of branch orientation on tree architecture and light absorption, the molecular mechanisms controlling this orientation are not well understood.

One of the key players in directing growth is the hormone auxin, whose uneven distribution causes the plant to change its direction of growth, branching, or bending with or against gravity (Moore 2002; Rakusová et al. 2011). At the molecular scale, a mutation in *WEEP* causes the weeping appearance of peach trees (*Prunus persica*), affects branch patterns in plum trees (*Prunus domestica*), and alters root systems in *Arabidopsis thaliana*, indicating its universal role in plant growth and response to gravity (Hollender et al. 2018; Kirschner et al. 2021; Johnson et al. 2022). WEEP homologs are involved in cell wall synthesis, participating in xylan methylation and lignin production (Guo et al. 2023), which suggests their role in affecting structural support.

In this issue of *Plant Physiology*, Kohler et al. (2024) explored the molecular mechanisms through which *WEEP* controls branch phenotype in peach. Through RNA sequencing performed on standard and weeping varieties of peaches, the authors revealed inverted expression of *SMALL AUXIN-UP RNAs* (*SAURs*), early-auxin response genes essential for cell elongation. Contrary to standard varieties, where *SAUR*s are upregulated on the lower side of shoots, weeping varieties exhibit increased *SAUR* concentration on the upper side, highlighting the importance of auxin gradients for orienting growth through the inhibition of PP2C-D phosphatases and activation of H^+^-ATPases (Spartz et al. 2014). The mislocalization of auxin and subsequent *SAUR* expression supports the Cholodny-Went hypothesis for gravitropism (Moore 2002; Rakusová et al. 2011), suggesting that the weeping phenotype results from altered auxin gradients (Fig. 1).

**Figure 1.**
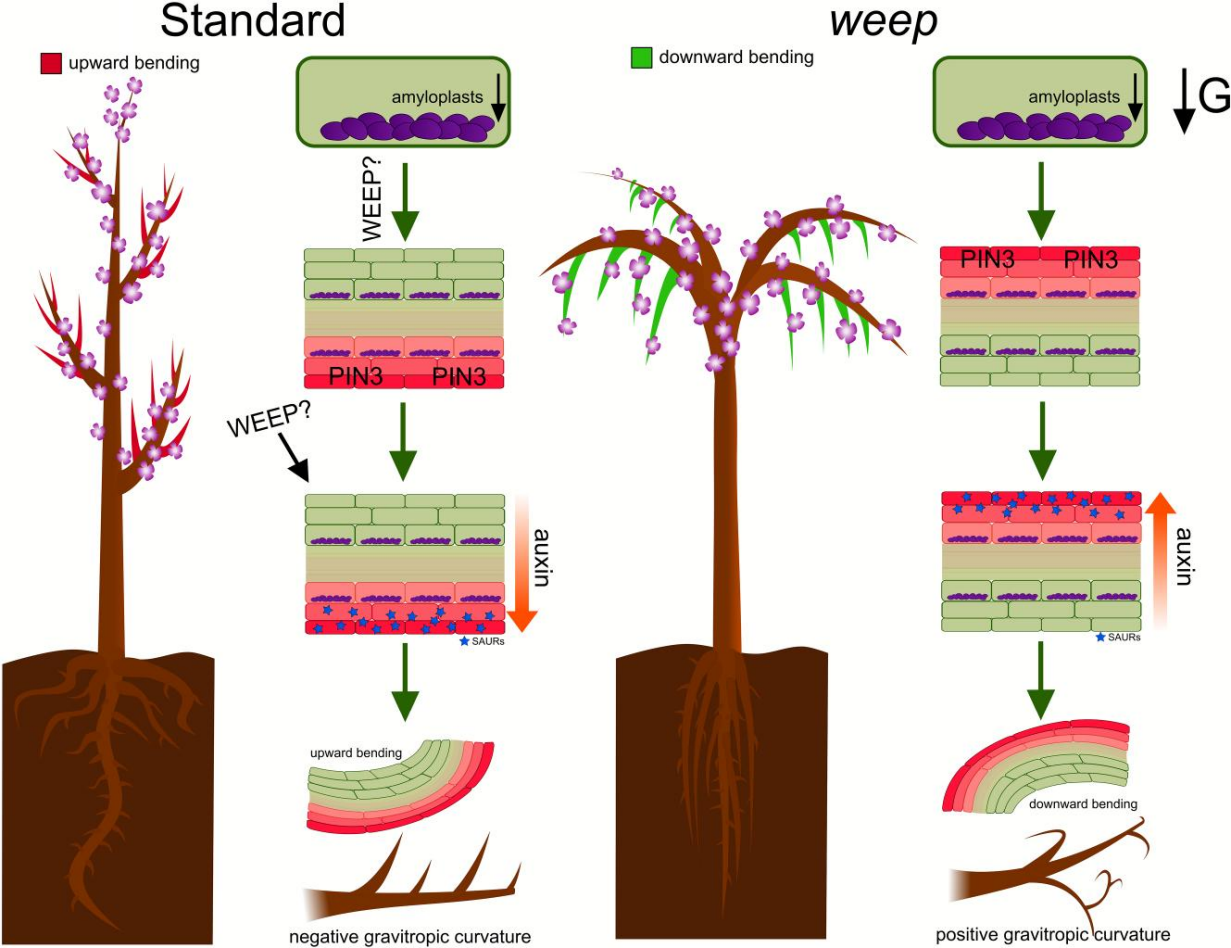
Gravitropic responses in standard and *weep* peach tree varieties influenced by *WEEP*. On the left, the standard variety is depicted with branches bending upward and roots growing downward, the lateral roots extending horizontally. Amyloplasts settle on the lower side of cells, signaling for PIN3 transporters to localize at this same side. This localization, potentially guided by WEEP, creates an auxin gradient, resulting in the accumulation of SAURs on the lower side, which then leads to cell elongation and the upward curvature of the branches, indicative of a negative gravitropic response. On the right, the *weep* variety is shown with branches bending downward and roots exhibiting a more vertical growth with sharply angled lateral roots. Despite normal amyloplast sedimentation due to gravity, the lack or malfunction of WEEP is hypothesized to invert PIN3 localization to the upper side, generating an inverted auxin gradient with SAUR accumulation at the top. This leads to cell elongation on the upper side, downward bending of branches, and a positive gravitropic curvature, characteristic of the distinct gravitropic behavior in *weep*.

The authors also identified the upregulation of *FASCICLIN-LIKE ARABINOGALACTAN-PROTEINS* (*FLA*s), which are involved in the regulation of cell wall synthesis. This process involves changes in cellulose microfibril angles and increases in arabinogalactan and cellulose content, essential for tension wood formation on the upper side of the branch when WEEP is functional. Like the altered *SAUR* expression pattern in *weep* mutants, *FLA*s showed increased activity on the lower side, suggesting a role in the downward growth of the branches. This observation aligns with studies that show tension wood formation in young shoots is associated with increased activity in genes regulated by auxin, ethylene, and gibberellic acid. Specifically, these genes become more active on the lower side of the shoot, opposite to the typical location for tension wood formation (Gerttula et al. 2015). These findings highlight the need to study auxin flux not just in epidermal cells but also in the vascular cambium, where xylem development and tension wood formation occur. This approach could show how auxin distribution affects growth and wood formation in young shoots, thus explaining the weeping growth pattern.

In contrast to the altered positive gravitropism in the *weep* shoot branches, the lateral roots displayed enhanced positive gravitropism, a phenomenon regulated by WEEP through its crucial role in enhancing polar auxin transport. This regulation leads to distinct root growth patterns, including reduced lateral root angles and a more vertical orientation, diverging from the upward root growth seen in *LAZY* mutants and arising from inverted auxin gradients. The importance of auxin distribution in root gravitropism is highlighted, with weeping peach roots exhibiting an enhanced positive gravitropic response that suggests a different mechanism of auxin action, possibly without reversing the auxin gradient. The distribution of PIN3 and PIN7 proteins, vital for auxin transport in root cells, is important for maintaining this gravitropic behavior. PIN3 typically localizes to the lower side of a cell, helping in directing root growth downward. Meanwhile, the positioning of PIN7 varies depending on the root type (Roychoudhry et al. 2023). This precise localization influences auxin flow and significantly affects root architecture and gravitropic responses.

In summary, the study by Kohler et al. (2024) offers novel insights into how plants respond to gravity, suggesting that WEEP plays a central role in guiding gravitropism in peach shoots and roots by influencing polar auxin transport. Mutations in *WEEP* result in unusual positive gravitropism in shoots, causing branches to grow downward, alongside an enhanced positive gravitropic response in roots. The weeping phenotype together with analyses of auxin-response gene expression indicates that WEEP is essential for the proper establishment of a gravitropic auxin gradient, a gradient that is reversed in weeping phenotypes. The authors proposed that WEEP alters the distribution of PIN proteins, especially PIN3, within the membrane of gravity-sensing statocytes. This adjustment in PIN protein localization is crucial for the regulation of auxin flow in response to gravity, affecting the localization of SAURs and subsequent cell elongation, which contributes to the bending toward or away from the gravity vector. Further research could focus on WEEP’s precise mechanisms, including its possible impact on PIN3 localization and overall regulation of auxin transport in response to gravity. This hypothesis is supported by WEEP localization and interaction patterns, suggesting a broad regulatory role in auxin homeostasis and transport critical for plant orientation. Additionally, the lack of a similar shoot phenotype in Arabidopsis *weep* loss-of-function mutant points to the need for further investigation into how FLAs contribute to lignification and the localization of tension wood, which may play a critical role in the development of the weeping phenotype.

## Dive Curated Terms

The following phenotypic, genotypic, and functional terms are of significance to the work described in this paper:

PIN7 Gramene: AT1G23080
PIN7 Araport: AT1G23080

PIN3 Gramene: AT1G70940
PIN3 Araport: AT1G70940
